# Clinical Reasoning in Forensic Psychiatry: Concepts, Processes, and Pitfalls

**DOI:** 10.3389/fpsyt.2021.691377

**Published:** 2021-08-05

**Authors:** Natalia Widiasih Raharjanti, Tjhin Wiguna, Agus Purwadianto, Diantha Soemantri, Saptawati Bardosono, Elizabeth Kristi Poerwandari, Marlina S. Mahajudin, Adhitya Sigit Ramadianto, César A. Alfonso, Ardi Findyartini, Nadia Rahmadiani Nugrahadi, Muhammad Qolby Lazuardi, Priscilla Aya Maheswari Subroto, Olivia Jeany Darmawan Adji Saroso, Monika Kristi Levania

**Affiliations:** ^1^Doctoral Program in Medical Sciences, Faculty of Medicine Universitas Indonesia, Jakarta, Indonesia; ^2^Department of Psychiatry, Dr. Cipto Mangunkusumo General Hospital, Faculty of Medicine Universitas Indonesia, Jakarta, Indonesia; ^3^Department of Forensic and Medicolegal, Dr. Cipto Mangunkusumo General Hospital, Faculty of Medicine Universitas Indonesia, Jakarta, Indonesia; ^4^Department of Medical Education, Faculty of Medicine Universitas Indonesia, Jakarta, Indonesia; ^5^Department of Nutrition, Dr. Cipto Mangunkusumo General Hospital, Faculty of Medicine Universitas Indonesia, Jakarta, Indonesia; ^6^Faculty of Psychology, Universitas Indonesia, Jakarta, Indonesia; ^7^Department of Psychiatry, Airlangga University, Surabaya, Indonesia; ^8^Department of Psychiatry, Columbia University, New York, NY, United States

**Keywords:** forensic psychiatry, psychomedicolegal analysis, clinical reasoning, cognitive bias, hypothetico-deductive model, illness-script theory, dual process theory, debiasing strategy

## Abstract

Forensic psychiatrists are often sought by the court of law to provide professional opinion on specific legal matters that have a major impact on the evaluee and possibly society at large. The quality of that opinion and recommendations rely on the quality of the analysis from the assessment results conducted by the psychiatrist. However, the definition and scope of a forensic psychiatric analysis is not clear. While existing literature on forensic psychiatric analysis generally includes organizing information, identifying relevant details, and formulating a set of forensic psychiatric opinions as components, there is no explicit and unified definition of these terms and process. This lack of clarity and guidelines may hinder forensic psychiatry from achieving its goal of providing objective information to the court or other relevant parties. Forensic psychiatric analysis exhibits numerous parallels to clinical reasoning in other fields of medicine. Therefore, this review aims to elaborate forensic psychiatric analysis through the lens of clinical reasoning, which has been developed by incorporating advances in cognitive sciences. We describe forensic psychiatric analysis through three prominent clinical reasoning theories: hypothetico-deductive model, illness script theory, and dual process theory. We expand those theories to elucidate how forensic psychiatrists use clinical reasoning not only to diagnose mental disorders, but also to determine mental capacities as requested by law. Cognitive biases are also described as potential threat to the accuracy of the assessment and analysis. Additionally, situated cognition theory helps elucidate how contextual factors influence risk of errors. Understanding the processes involved in forensic psychiatric analysis and their pitfalls can assist forensic psychiatrists to be aware of and try to mitigate their bias. Debiasing strategies that have been implemented in other fields of medicine to mitigate errors in clinical reasoning can be adapted for forensic psychiatry. This may also shape the training program of general psychiatrists and forensic psychiatrists alike.

## Background

Forensic psychiatry is a subspecialty within psychiatry that addresses the interface between mental health and the law, including how people with mental health conditions interact with legal systems (1). Conducting forensic psychiatric evaluations and conveying the results, through written report or oral testimony in court, make up a major part of forensic psychiatric practice. Forensic psychiatrists are often sought to assist the court in answering specific legal questions by providing a professional opinion. The quality of that opinion, as the final product of a forensic psychiatric evaluation, relies on the quality of underlying examination and analysis (2).

Forensic psychiatric analysis forms the assessing psychiatrist's opinions and recommendations, which are arguably the most important part of a forensic psychiatric report (3). It generally includes organizing information acquired during examination, identifying relevant details, and building a formulation to answer the legal question. It is a complex task at the intersection of psychiatry and psychology, medicine, and the law. The process requires data from the examination as input, and then as output, produces a report containing expert opinion regarding the case. In many aspects, the forensic psychiatric analysis is comparable to clinical reasoning in other fields of medicine. It requires judicious use of various cognitive and metacognitive skills to make sense of the wealth of information acquired during the examination to come to a conclusion (4).

Consequently, the quality of a forensic psychiatry analysis is critical because the report will be taken into consideration in the court of law. The psychiatrist's opinion contributes in shaping the legal decision that will impact the evaluee for a long time, potentially altering the course of their life (5). An ideal forensic evaluation is mainly focused on answering the legal question posed to the psychiatrist, in contrast to the patient's welfare in clinical psychiatry. While there is considerable debate about ethics in forensic psychiatry (6–8), psychiatrists conducting forensic psychiatric evaluations should be aware of its wide-ranging implications to the evaluee, the psychiatrist, and society in general. A forensic psychiatric evaluation of poor quality may potentially lead to miscarriage of justice, present safety risk for individuals and society, and put the psychiatrist at risk of legal conflict (9, 10).

Forensic psychiatric evaluations are conducted by psychiatrists in all parts of the world, although technical details may vary according to the local legal and psychiatric landscape. For example, Indonesian law explicitly states “psychomedicolegal analysis” as a mandatory step in conducting forensic psychiatric evaluation, along with more familiar steps such as psychiatric interview and psychometric testing. However, it does not provide a clear definition of and reference for “psychomedicolegal analysis.” This ambiguity may lead to different interpretations of what is expected from and the limitations of forensic psychiatrists. In practice, it may contribute to miscarriage of justice and risk of legal conflicts between evaluees and psychiatrists. This is also a serious issue even in jurisdictions that do not explicitly acknowledge psychomedicolegal or forensic psychiatric analysis.

An elucidation of forensic psychiatric analysis is important and beneficial for psychiatrists and service users alike. This review aims to elaborate on forensic psychiatric evaluation, especially its analysis, through the lens of clinical reasoning. We seek to identify parallels between the two processes as well as their shared potential for errors. This is a promising approach, given the advances in cognitive sciences underlying clinical reasoning. As this is one of the first, if not the only, literature attempting to bridge forensic psychiatry and clinical reasoning, this can also serve as a foundation for further research in the field. Moreover, it will contribute to shaping the training of forensic psychiatry at all levels, and foster exploration of possible avenues for remediation of potential shortcomings.

## Theories of Clinical Reasoning

Basic and clinical knowledge is important and necessary to practice medicine and its specialties, including forensic psychiatry. However, knowledge alone is not sufficient. Clinicians also need to know how to organize and utilize that knowledge in order to care for patients (11, 12). Thus, clinical reasoning is an essential skill of a clinician. Clinical reasoning in its broadest meaning refers to all processes of knowing and doing by clinicians directly involved in patient care, encompassing the formulation of a working and differential diagnosis, treatment, and prognosis (13). A considerable proportion of literature in clinical reasoning is focused on diagnostic reasoning, which will also be the focus of this review.

As reasoning is a domain of cognitive function, clinical reasoning literature has increasingly relied on concepts from cognitive sciences (14). Three of the most prominent theories of clinical reasoning will be discussed here: the hypothetico-deductive model, illness script theory, and dual process theory (15). In these cognition-oriented theories, the human emotion is considered as a factor that may influence the cognitive processes involved in reasoning (16, 17).

### Hypothetico-Deductive Model

The hypothetico-deductive model was one of the earliest attempts to describe the clinical reasoning process (18, 19). According to this model, clinicians continually generate diagnostic hypotheses about their patient. The generation of diagnostic hypotheses starts from the initiation of the patient encounter when the clinician only has little information. They then deduce the logical consequences of those hypotheses. Those deductions are tested through further investigations, so that the clinician can come to a diagnostic decision by accepting or rejecting their hypotheses (20). While it cannot fully explain the complex nature of clinical reasoning, this model is a useful representation of at least some of its cognitive processes. In its original formulation, the hypothetico-deductive model does not differentiate between the clinical reasoning process of a novice and an expert. It was later proposed that the difference lies on the quality of the hypotheses: experts generate stronger hypotheses so that the testing phase becomes more efficient (15, 21, 22).

### Illness Script Theory

Illness script theory grew from the concept of schema, the basic unit that is used to remember the essence or a modicum of knowledge (23, 24). Scripts are high-level, conceptual structures of knowledge which represent general event sequences. The events in a script may share temporal, causal, or hierarchical connections. Besides fixed general information, scripts also have variables that can be filled in for a particular situation. When a script is activated and its variables are filled with incidental information, it is said to be instantiated (23).

In this theory, illnesses are understood as a sequence of events reflecting the general manifestation of a disease. The three main components of an illness script are the *Enabling Conditions*, factors that determine the probability of a certain disease; the *Fault*, pathophysiology of the disease; and the *Consequences*, the manifestation of the disease (23). Illness scripts can be described as list-like structures containing a clinician's expected findings in a disease (15). These scripts are generated by repeated direct experience and stored in long-term memory (23). Training setting, local epidemiology, sociodemographic characteristics of local population, and geography can influence the types of experiences a clinician encounters, which in turn determines what illness scripts are available to them (25).

Upon a patient encounter, initial information activates one or more available illness scripts. Because scripts can predict sequence of events, it also directs how the clinician will approach a case. As patient information accumulates, scripts matching the patient's characteristics are reinforced, while less relevant scripts are attenuated or even dismissed. The most likely diagnosis is the one script that shares the most characteristics with the patient. If the patient does not fit any script adequately or fits too many scripts at the same time, more deliberate reasoning is needed (26).

Through experience, expert clinicians accumulate a larger number of illness scripts and may emphasize different components of the scripts themselves. Novice clinicians put more emphasis on the *Fault* of a script, and their script may not yet be structured for practical applications. In contrast, experts diagnose a patient using more of the *Enabling Conditions* and *Consequences*, which are instantiated relatively early (23). Experts are also more capable of identifying salient information, resulting in faster and more suitable script activation, while novices may face difficulties filtering out clinically irrelevant information (24). With the accumulation of experience, illness scripts can be activated without conscious awareness, relying on pattern recognition instead (24).

### Dual Process Theory

Dual process theory (DPT) was first developed in the field of cognitive sciences. This theory was then later adapted to medicine as it may help elucidate the different processes clinicians use to reach a decision (15, 27). According to the theory, any cognitive task can activate two forms of processing (28). The DPT literature often refers to System 1 and System 2 processing modes. System 1 is described as non-analytic, fast, and intuitive; while System 2 is analytic, deliberate, and logical. However, proponents of DPT itself have criticized such simplistic description and offered a revised explanation, including changing the terms to Type 1 and Type 2 processing (28, 29).

Type 1 processes are characterized by their autonomy, automatically activated when relevant stimuli are encountered. It is not reliant on higher-order cognitive control and does not deplete working memory capacity. This level of autonomy correlates to faster rate of processing and utilization of associative learning. It does not imply that Type 1 processing follows no rules, but rather that the rules have been made implicit through repeated practice or overlearning (28). In clinical reasoning, Type 1 processes are associated with heuristics and mental shortcuts to arrive at a decision using minimal effort (30). Pattern recognition may be one of the most common forms, or even the basis, of Type 1 processing. Clinicians unconsciously recognize the pattern of a patient's clinical presentation by matching it with patterns already stored in long-term memory. (31). While Type 1 processing may seem distant from rational and careful clinical reasoning, it is capable to arrive at the right answer quite frequently. This is especially true for clinicians encountering patients with typical disease presentations. Conversely, it is prone to fail when the clinician encounters atypical or overlapping presentations (30).

Type 1 processing is frequently used in clinical practice because of its tolerance toward uncertainty. It starts generating hypotheses as soon as initial information is obtained, and, according to the concept of “bounded rationality,” will try to reach a sufficiently-informed decision even in less than ideal circumstances, such as incomplete information or limited resources (30). However, Type 1 processing can be modified by influences that is outside the clinician's conscious awareness, such as patient and clinician characteristics, illness presentation, and situational factors. Thus, this type of reasoning is shaped by clinical experience in its broadest sense, not only the experience of formulating a diagnosis, but also the experience of interacting with the patient and their family, managing work pressure, and many more (30).

On the other hand, Type 2 processing is characterized by the engagement of working memory and many other higher-order cognitive functions, which are correlated to general cognitive ability. This characteristic also leads to its other associated features, such as slower but more meticulous reasoning (28)^.^ Another key feature of Type 2 processing is the cognitive decoupling of primary and secondary representations that allow for mental simulations and hypothetical thinking (28).

Type 2 processing is activated when clinicians encounter novel cases from which no pattern or script can be readily discerned. Instead, it arrives at a decision through normative and rational reasoning process based on established rules (30). Diagnostic hypotheses are systematically and analytically tested before deciding on the most likely diagnosis. Thus, it is said that hypothetico-deductive thinking forms the basis of Type 2 processing (31). Decisions made through Type 2 processing are robust and logically valid. Yet, it does not guarantee that the decision is logically sound, i.e., it can produce an incorrect decision when the input is inaccurate. For example, failure to elicit depressive symptoms in a person presenting with psychosis will lead a psychiatrist to “correctly” diagnose schizophrenia instead of schizoaffective disorder. Nevertheless, Type 2 processing is often associated with higher probability of accurate decision compared to Type 1, but it also requires more cognitive capacity (30).

Clinical reasoning in daily practice involves both Type 1 and Type 2 processing, and the combination of strategies is thought to be superior to either strategy alone (32). When the clinician receive initial patient information, such as presenting symptoms, clinical signs, and important patient characteristics, Type 1 processing will be instantly and unconsciously engaged. If a matching pattern already exists in their memory, they will be reflexively recognized. This pattern recognition will serve as the basis of their diagnostic decision. For example, a complaint of confusion and forgetfulness in an elderly person can be quickly suspected as dementia. According to Croskerry, if for any reason such pattern recognition does not occur, Type 2 processing will be activated to organize and make sense of the information (30). However, Pelaccia states that Type 2 analytic processing will always be engaged to confirm or refute the diagnostic hypotheses generated by Type 1 processing (31). As a consequence of this framework, the hypothesis formed early in the clinical encounter by Type 1 processing shapes the Type 2 processing as well, by directing the hypotheses to be tested by analytical reasoning. This concurs with the finding that early diagnostic hypothesis is usually carried to the end as working diagnosis (22).

The hypothesis-testing role of Type 2 processing can be described as monitoring and potentially overruling Type 1 processing (30, 33). For example, a working diagnosis made through pattern recognition will be reassessed if an atypical finding is found. Type 2 processing takes over to analyze it before confirming or refuting that diagnosis. Conversely, Type 1 processing may interfere with the logical processes of Type 2, as often happens when a clinician decides to follow their intuition rather than clinical guidelines. While this may prove useful in a handful of cases, generally it will reduce diagnostic accuracy (30). A review found that clinicians are more likely to utilize analytic reasoning if adequate time is available, the outcome entails significant risk, or the situation is complex, ambiguous, and uncertain (31).

Nevertheless, many processes cannot be mapped neatly as Type 1 or Type 2, and the characteristics of each are not clear-cut. Cognitive continuum theory puts intuition and analysis not as separate systems, but as poles on a continuum. In the extreme intuitive pole lie processes such as intuition and pattern recognition. On the other end of the continuum are algorithms. A reasoning process is said to be analytical if every step in the process is justifiable and retraceable. The degree of justifiability and retraceability determines where a certain “quasirational” process is located on the continuum. Cognitive tasks can also be mapped out in the continuum to match the required type of reasoning (34).

## Clinical Reasoning in Forensic Assessments

In a forensic psychiatric evaluation, the product of clinical reasoning is not only a psychiatric diagnosis. The assessing psychiatrist must also “diagnose” the specific mental capacity of the evaluee to answer the legal question posed by the retaining party. To make that “diagnosis,” they need to report the examinee's relevant mental state or level of functioning, medical diagnosis, and how they relate to each other and to legal standards applicable to the case. It is imperative that the evaluation process carefully considers all of these aspects (2, 35). The theories of clinical reasoning described in the previous section can serve as useful framework to understand forensic psychiatric analysis.

In the perspective of hypothetico-deductive model, the psychiatrist will make various hypotheses about the evaluee throughout a forensic psychiatric examination. From those hypotheses, they then make deductions based on their prior knowledge of legal standards. These deductions will be tested through the interview or other examination methods so that the psychiatrist can confirm or reject their hypotheses. This is an iterative process that repeats until the psychiatrist has made all the relevant diagnoses (psychiatric, medical, legal).

*Case vignette. Doctor M is conducting a forensic psychiatric evaluation to determine whether Mrs. S is competent to stand trial for her murder charges. Upon learning that Mrs. S had been diagnosed with schizophrenia and had not been adequately treated, Dr. M hypothesizes that she does not have the capacity to fully participate in her defense during trial. Using her knowledge of relevant laws, Dr. M deduces that if Mrs. S is indeed incompetent to stand trial, she would not understand the charges brought against her and that she cannot identify the parties involved in her trial. Subsequently, Dr. M tests her assumptions by eliciting what Mrs. S understands about her predicament in the forensic interview. The information gained through her interview ultimately confirmed her hypothesis, which she narrates in her report*.

Similar to illness script theory in its original formulation, information acquired from forensic psychiatric examination is used to instantiate activated scripts. However, scripts in forensic psychiatric evaluations also contain legal principles that needs to be instantiated as well, expanding them into “forensic scripts” as psychiatrists accumulate experience of conducting forensic evaluations. Forensic scripts are shaped by prevailing legal standards; thus, scripts for the same mental capacities may differ according to local jurisdiction.

*Case vignette. When conducting forensic evaluation on Mrs. S, both the competent and incompetent to stand trial scripts are activated in Dr. M's mind. In this case, the Enabling Condition may be untreated schizophrenia or crystallized delusion of grandiosity, as these characteristics influence Mrs. S's mental capacity. The Fault or underlying “pathophysiology” could be impaired reality testing or general cognitive impairment, with the Consequences that Mrs. S cannot fully participate in the trial and her own legal defense. Those are the variables that need to be instantiated throughout the examination process. The final decision will come to which script is more strongly reinforced by available information*.

Considering the impact of forensic psychiatric reports on an evaluee's life, it is rather expected that assessing psychiatrists make full use of Type 2 analytic processes to reach a logical, accurate decision. However, Type 1 non-analytic processing still play a significant role in forensic psychiatric analysis. In fact, its involvement may be inevitable, as it is automatically activated by relevant stimuli. This is in line with the framework that Type 1 processing provides initial hypotheses for Type 2 processing to analyze. Heuristics and other mental shortcuts are used to minimize cognitive load in the complex analysis of forensic cases, and they may correctly direct the evaluation. However, those non-analytic processes are also error-prone, especially when the case does not correspond with, but is then “forced” into existing heuristics (36, 37). Hence, it is important that Type 2 analytic processing prevent such errors by carefully analyzing the details of the case and revising the diagnostic hypothesis as necessary. Type 2 override of Type 1 processes is preferrable and necessary for the psychiatrist to conduct an accurate and comprehensive assessment.

*Case vignette. After seeing Mrs. S for a few minutes, noting her unkempt appearance, Dr. M immediately thought that she is incompetent to stand trial. However, Dr. M remembers that the diagnosis of schizophrenia and the consideration of competency to stand trial does not rely solely on the evaluee's appearance. Therefore, she begins conducting deeper interview to satisfy the diagnostic criteria and legal standards*.

Another important characteristic of a forensic psychiatric evaluation is that results must stand scrutiny in court, whether by the judge or opposing party. Each opinion the psychiatrist puts forward in the report must be based on information from the examination process, and the forensic analysis must be clearly delineated in the report. There should be tight consistency between the data, reasoning process, resulting opinions, and recommendations (2, 3). Furthermore, the chain of reasoning must be written clearly and in plain language. The report should be understandable to laypeople, as most people involved in the case do not have medical or forensic science educational or clinical background (38). This requirement for the reasoning process of a forensic psychiatric analysis to be explicitly justified and retraceable is consistent with the characteristics of a Type 2 process. In contrast, the process to reach a conclusion through a Type 1 processing cannot be described to an outside observer, even when the conclusions themselves are accurate (34).

To assist novice and expert psychiatrists alike, there are general rules and practice guidelines for different types of forensic psychiatric evaluation in criminal and civil law cases (2, 39–41). For example, psychiatrists conducting an insanity defense evaluation must determine the defendant's mental state at the time of the crime, its relationship to the criminal behavior, and whether it meets legal standards of insanity in that jurisdiction. With experience, the psychiatrist may encounter cases with similar issues, such as insanity defense evaluations for persons living with schizophrenia. The examinees may even show similar symptoms, such as command auditory hallucinations. The legal question and standards are identical, and the examination process will be largely similar. Repeated practice on the same set of clinical reasoning tasks can contribute to the development and refinement of forensic scripts and Type 1 heuristics (42). It will help psychiatrists to identify salient information from less relevant data, and to activate relevant scripts more readily.

Nevertheless, evaluees with similar psychiatric and legal issues may have very different developmental history, social context, and chain of events that lead to their alleged behavior. Those aspects must be taken into account in the analysis, which may lead to vastly different conclusions and recommendations. It can be reasonably said that no two cases are the same; hence, each case requires individualized analysis. Consequently, it is crucial that assessing psychiatrists do not rely too heavily on Type 1 processes that may lead to inaccurate conclusions. As Type 1 processing will inevitably generate diagnostic hypotheses in every case, it is crucial that psychiatrists deliberately employ Type 2 processing to analyse the finer details of the case in order to reach more accurate conclusions.

When conducting a forensic psychiatric evaluation, psychiatrists must navigate the challenging interface of psychiatry and the law. Legal decisions are largely categorical, e.g., either the evaluee can be held responsible for their alleged offense or not, either the evaluee is impaired enough to need guardianship or not. These categories are defined by the letter of law, and do not necessarily have parallel psychological categorizations. While psychiatric diagnoses are categorical, the mental state or psychological functions that determine mental capacity is dimensional. For example, cognitive impairment, reality testing ability, or appreciation of the nature of an offense exists in a wide spectrum. Psychiatrists do need to understand the applicable legal standards in each specific case in order to direct their clinical reasoning. Nevertheless, mental capacity will ultimately be decided by the court, who uses the information contained in forensic psychiatric report to come to a legal decision. Psychiatrists are advised to provide detailed information that is necessary for the court, but to refrain from coming to the legal conclusion themselves (5).

In summary, through processes similar to clinical reasoning, the psychiatrist must be able to integrate their prior knowledge of clinical and forensic psychiatry with current and actual information from the case at hand to achieve the objectives of the assessment, while considering the whole context of the evaluation and anticipating possible consequences (43).

## Errors in Clinical Reasoning and Forensic Psychiatric Analysis

The growing literature on clinical reasoning is followed by a deeper understanding of how errors in reasoning can happen. Errors in clinical reasoning, especially in diagnosis, are a major issue in medicine and pose a significant threat to patient safety. Clinicians, educators, administrators, and other stakeholders have made serious efforts to understand how the clinical reasoning process can go wrong and what factors influence those errors (44). Similarly, errors in forensic evaluations have also gained attention. A survey of forensic mental health professionals from 39 countries found that 79% of them believe that bias is a concern in their field (45). Several papers have also addressed this issue by proposing various methods to identify and mitigate bias in forensic evaluations (46–48). As clinical reasoning theories can assist in elaborating forensic psychiatric analysis, understanding errors in clinical reasoning may also help elaborate on forensic analysis errors.

There are different sources of error in clinical reasoning. Graber classified diagnostic errors into no-fault errors, system errors, and cognitive errors (49). Although the classification is based on findings in internal medicine, it can can inform classification of errors in forensic psychiatric assessment, as both are specialties in medicine and thus share similarities in their clinical reasoning (50–52). Errors are considered no-fault if no reasonable clinician could have identified the diagnosis. They could be due to lack of access to patient information or extremely atypical presentation. For example, a psychiatrist may not know that an evaluee had experienced a previous psychotic episode if the evaluee is still experiencing significant psychotic symptoms that impairs their communication and no other source of information is available. System errors are caused by organizational issues and inadequate resources, such as poor workplace environment or equipment failure. Psychiatrists working in rural areas with limited radiology services may fail to ascertain the diagnosis of mental disorders due to brain lesions. Last, cognitive errors may be the result of a knowledge gap, faulty data gathering, or faulty processing of information (49, 53). In the literature, the terms “cognitive errors” and “cognitive bias” are used more narrowly to refer to faulty information processing (29). The study by Graber also showed that diagnostic errors are more likely to be caused by cognitive errors rather than insufficient knowledge (49).

The idea that diagnostic errors may have different causes are echoed by Croskerry, who attributes diagnostic errors to *dysrationalia* (27). It is divided into two categories: processing problems and content problems. In this model, processing problems are rooted in the cognitive architecture of the human brain and is related to the concept of cognitive miserliness, which assumes that the brain always seeks to minimize cognitive effort to solve a problem. This may cause clinicians to jump into inaccurate conclusions, as the information gathering process is not broad or deep enough, and what little information is gained from that inadequate process is accepted at face value. Content problems are caused by problems in the “software” of the brain, also termed *mindware*. Errors happen when the mindware have gaps of knowledge or is contaminated. Knowledge gaps exist where the information needed for reasoning is not available, either because it is not yet acquired or it has been forgotten. On the other hand, mindware contamination is related to cognitive and affective biases (27).

Various kinds of cognitive errors that affect clinical reasoning have been described in the literature, and they are somewhat related to each other (49, 54–56). For example, *confirmation bias* leads clinicians to prioritize information that supports their initial hypothesis, to the point that they ignore evidence that points to the opposite, and *anchoring bias*, meaning that clinicians become rigidly anchored to a certain diagnostic hypothesis early on, not modifying it in the face of new information (56). Clinicians with availability bias would judge the probability of a diagnosis based on how readily it comes to mind. When clinicians “confirm” a diagnosis too early with insufficient evidence, they may be committing an error of premature closure (57). Lastly, a systematic review found that overconfidence, the feeling that one knows more than what they actually do, is the most common cognitive bias leading to judgement errors (58).

As DPT asserts that Type 2 processing monitors Type 1 processes to correct it when an error or bias is detected, those cognitive errors happen when Type 1 processing generates an erroneous hypothesis and Type 2 processing fails to detect and modify it (31, 33). Thus, it has become clear that the monitoring function of Type 2 processing is not failproof, or as Kahneman put it, the corrective thoughts of Type 2 processing is not always accessible, in contrast to Type 1 heuristics that are easily accessible (33). This Type 2 processing failure may be associated with personal factors such as overconfidence, complacency, and lack of motivation, or with contextual factors such as time restriction, multi-tasking, sleep deprivation, and distraction (31, 33). It is also related to metacognitive knowledge: individuals are less likely to correct their intuition if they are unaware that they are using heuristics (33). Last, the monitoring function of Type 2 processing may simply be inhibited by the intuitive Type 1 processing (31). Nevertheless, Type 2 processing may still come to an erroneous conclusion, especially when the clinician lacks the necessary knowledge or information. In fact, with such knowledge gaps, Type 2 override of Type 1 processing may introduce errors (36).

The source of cognitive errors or bias can be found in almost all layers of a forensic psychiatric evaluation. According to a taxonomy by Dror, there are eight sources of bias, organized into three categories, that can impact decision-making in the forensic sciences (59, 60). This taxonomy is sorted into tiers, reflecting the scope of their influence from general to case specific.

The first category at the base is the cognitive architecture that all humans share. Various limitations have shaped how the human brain receive and make sense of information. This is parallel to the processing problem in the dysrationalia framework by Croskerry (27). In short, the brain does not “record” and “playback” the world like a video camera. As the cognitive miser assumption asserts, the brain uses different processes to make information processing more efficient with as little cognitive load as possible (42). This shared nature makes it a general influence, in the sense that it happens regardless of the case or the assessing psychiatrist.

In the second category are sources of bias that arise from each psychiatrist as a person: their personality, background, education and training, as well as working environment (59, 60). Education and training experience, especially during residency and fellowship, may impart different theoretical and practical orientations when conducting forensic psychiatric evaluations, and shape psychiatrists' approaches to solve problems and cope with the pressure of forensic psychiatric work. It can also affect base rate expectations of examination findings. A psychiatrists' upbringing and personality can determine their values and motivations as well as their tolerance to risk and uncertainty that is almost always present in forensic psychiatric cases (61, 62). Empathy is also known to influence how forensic evaluators perceive their evaluee (62, 63). The working environment may impact clinical reasoning through various pathways, such as the adversarial legal system, workplace culture, targets, and the physical environment of the workplace (64–66). The influence of personal factors can still be detected even when evaluators use structured tools (67, 68).

In the last category, the sources of bias are related to the specific case that is being worked on by the psychiatrist (59, 60). Bias may be caused by the case information itself, especially due to the nature of forensic psychiatric evaluations that mostly require extensive interviews and interaction with the evaluee. The reference materials, through which the psychiatrist interpret their findings, can also bias their conclusions. Contextual information, even those irrelevant to the case and legal question, can influence how the assessing psychiatrist collect, organize, and interpret case information. For example, widespread media attention and extensive news coverage of a criminal case may unsconsciously nudge an assessing psychiatrist to look for information that confirms prevailing attitudes toward the defendant and to ignore conflicting findings.

As bias in forensic evaluations and clinical reasoning has been elaborated mostly through the perspective of cognitive sciences, the influence of emotion is relatively less discussed. Nevertheless, emotion has been identified as a modifier of the cognitive processes in clinical reasoning and decision-making, including in forensic psychiatric evaluations (47). In their review, Lerner put forward different ways emotions can influence decision-making processes. They influence decisions by shaping the content of thought, the depth of processing, or activation of certain goals (69). Thus, it is not surprising that clinicians and clinicians-in-training had traditionally been advised to detach from their emotions and maintain emotional neutrality (70). Emotions experienced before or during clinical reasoning impacts performance, such as time required to reach diagnostic closure and diagnostic accuracy (16, 71). The effect of emotions as part of contextual factors in clinical reasoning can be identified in medical students, resident physicians, and medical experts (72–74). Nevertheless, when utilized judiciously, emotions can facilitate forensic psychiatric assessment through improved rapport and understanding between evaluee and evaluator (70, 75).

There can be multiple sources of emotion that may introduce bias in a forensic psychiatric evaluation. In the taxonomy of sources of bias by Dror, emotion can be found in several of them. During the course of a forensic psychiatric evaluation, the examinee and the details of their case may evoke positive and/or negative emotions in a process similar to countertransference in psychotherapy. *Forensic countertransference* has been defined as “all feelings, whether conscious or unconscious, that are evoked in forensic examiners during evaluation or testimony, in response to examinee and nonexaminee variables that have the potential to have an impact on the objectivity of their forensic opinions” (76). The definition acknowledges that the emotion can come from the psychiatrist themselves or from external factors. Moreover, the emotion may be integral to the decision-making itself or carried over from an unrelated situation (69).

The evocation of certain emotions is caused not only by the case material, but also by their interaction with personal factors of the assessing psychiatrist. Emotions can be associated with the psychiatrists' values and motivations, of which they may not be consciously aware. They may have a desire to help, need to show expertise, fear of legal complications, or other personal motivations. These motivations are, in turn, shaped by their upbringing and personality as well as educational and clinical experience. Additionally, emotion can also be provoked by any of the parties involved in the case (47). Furthermore, emotions are influenced by contextual factors beyond the psychiatrist and the evaluee, such as work environment, fatigue, resources, and cultural and social context. Lastly, emotions are also shaped by circadian and seasonal variations, physiological conditions, and mental health issues (16, 17).

Even though elaborating on cognitive and affective biases can help shed a light on how clinical reasoning errors happen, focusing solely on those internal cognitive processes will fail to paint a complete picture. Cognition is also influenced by the environment or context surrounding each individual (77). *Situativity theories* expand the traditional models of clinical reasoning to incorporate factors beyond the clinician, such as the patient, other people in the clinical encounter, the physical and sociocultural setting, and the interactions that occur among them. Consequently, they also contribute to the risk of committing errors (77). Cognition, including clinical reasoning, is never an isolated singular process. When the analysis shifts from the individual clinician to the environment and their interaction, it becomes clear that cognition is situated in its specific context. This is the central tenet of “situated cognition” (78). In fact, contextual factors may inadvertently influence a clinician to give different diagnoses for different patients who present with the same signs and symptoms due to the same illness. This phenomenon is termed “context specificity,” as opposed to “content specificity,” and has been experimentally proven to affect diagnostic accuracy (79).

With an understanding of situated cognition, an overlap between system error and cognitive error emerges (80). Clinical reasoning can be affected by situations that are commonly experienced in clinical practice: time constraints, task interruptions, administrative demands, and noisy or cramped work environment. For example, a generally competent clinician would be at a higher risk of diagnostic error when encountering a case with atypical presentation at the end of their shift after an especially exhausting day due to understaffing. These are circumstances that promote Type 1 processing as it places lighter cognitive load on the clinician. At the same time, they also negatively impact the monitoring function of Type 2 processing over the potential biases of Type 1 processing. Thus, unfavorable contextual factors may give rise to cognitive errors due to increased use of bias-prone Type 1 processing and impairment of Type 2 monitoring process (30).

## Case Illustration

Errors in analysis and their contributing factors do not occur in isolation, as illustrated in the following example:

Dr. S is an early-career psychiatrist working in a general hospital in Indonesia. During residency, she had a pleasant and productive rotation in forensic psychiatry, but she felt that her experience in civil law cases was rather lacking. As she has a keen interest in forensic psychiatry, she was glad to receive a referral for a fitness-to-work assessment. Without asking further details, she agreed to set up an appointment to conduct the evaluation and to produce a report within 2 weeks, as requested in the referral. She hoped the case would be challenging as an opportunity to develop her skills in forensic psychiatry even further. More specifically, she anticipated doing fitness-to-work assessments as a way to advocate for people living with mental health issues to gain meaningful employment.

The evaluee is Mr. D, a 27-years old man who works as a staff member in an accountant's office. His employer requests an evaluation because for several months Mr. D had been neglecting his work duties, and his colleagues reported that he had been speaking “strangely” when alone or with them. His medical record showed that he has a history of schizophrenia since his early-20's, with the first episode when he was attending college. He experienced auditory hallucinations and persecutory delusions that interfered with his daily activities. He underwent psychiatric treatment with the support of his family, and his condition was managed with antipsychotics and supportive psychotherapy. He had been hospitalized twice: the first time at the onset of his psychotic symptoms, and the second 5 years ago when he stopped taking his medications. However, he was able to resume treatment, finish college, and find stable work.

After reviewing Mr. D's records, Dr. S saw him as a victim of stigmatization, which is still rather common in Indonesian society. She is convinced that Mr. D has entered symptom remission and feels motivated to secure his employment so that he can achieve full recovery. Without realizing it, Dr. S had started forming her opinion about the evaluee before she even met him.

On the day of evaluation, Dr. S was rather focused on her own hypothesis. She was very intent on proving that Mr. D has entered symptom remission and is capable of continuing his job independently. She brushed aside Mr. D's unkempt appearance and his disordered thought process as residual symptoms that would not interfere with his work. She accepted Mr. D's assertion that he is doing fine and is able to perform well at work, reassured with what she read about his past progress in the medical record. Hence, she did not seek comprehensive information from Mr. D's superiors and co-workers. Furthermore, she did not think about confirming Mr. D's assertion through objective proof about his recent performance, such as attendance reports or written performance reviews. She felt satisfied with her findings and wrote her report, thinking that the requesting party would appreciate her fast pace in completing the evaluation.

This short case vignette shows how personal and contextual factors interact and may cause bias in forensic psychiatric analysis. Dr. S's experience during residency and personal desire to develop her skills motivated her to accept the request for a fit-to-work evaluation. However, her eagerness also led her to accept the 2-weeks deadline, putting a considerable time constraint on the evaluation. Her motivation to advocate for the employment of those living with mental disorders shaped her hypothesis that the evaluee was capable to continue to work but is being stigmatized by his workplace, even before she met the evaluee. This motivation, which may stem from past experiences, is not a problem in itself, but should be consciously recognized and mitigated to minimize its influence on her analysis.

Dr. S was rather anchored to her diagnostic hypothesis. During the actual examination, she focused on gathering information that supports the hypothesis and rationalized her dismissal of information that may prove otherwise. With the accumulation of evidence, although one-sided, she felt justified to confirm her hypothesis. Additionally, due to the time constraint, she did not seek information from co-workers or written performance reports. She felt confident conducting the evaluation, recalling the positive feedback she earned during her forensic psychiatry rotation. She considered her evaluation complete and wrote the report, not realizing that she had prematurely closed the case.

## Training for Clinical Reasoning and Mitigating Bias

Beyond the general consensus that clinical reasoning should be explicitly and deliberately included in medical education as well as its specialties and subspecialties, there are several approaches that can be undertaken to equip psychiatrists with the techniques to mitigate bias in forensic evaluations. A starting principle is that cognitive debiasing is not a one-off event. It takes different interventions to assist learners to be aware of bias, to commit to change, to learn debiasing strategies, and to implement them consistently (81). A systematic review found that strategies to improve critical thinking abilities, technological aid, and motivational strategies have been tried to mitigate bias. The majority of these debiasing strategies show some success, hinting at their usefulness (82).

Bias mitigation can begin early in the training period, from medical school to residency and fellowshp, to prevent bias in future decisions. It can take the form of didactics about clinical reasoning or integrated into other learning activities (81). As a consequence of DPT, training for clinical reasoning should aim to “train” Type 1 processing to produce more accurate hypotheses and to “strengthen” the corrective functions of Type 2 processing. Repeated exposure to similar forensic cases allows psychiatrists to abstract them into sharper illness and forensic scripts, while having a good variety of cases helps their analytic skills. Feedback techniques can be modified to foster psychiatrists' clinical reasoning and bias detection, such as by giving feedback on every step of the forensic psychiatric analysis instead of the finished report only (31, 83). This “serial-cue” approach (as opposed to “whole-case” approach) is appropriate, considering that psychiatrists have already formed illness and forensic scripts from their previous experience and they only need to refine them (84).

Debiasing interventions can also be done as the clinical reasoning process happens during the forensic psychiatric examination and analysis. They can directly aid decision-making, such as using statistical prediction rules or other support tools. Another approach is to force psychiatrists to pause and examine their reasoning, to check whether cognitive bias had inadvertently shaped their conclusions. These methods are introspective, asking the psychiatrist to reflect on their own reasoning process, as well as serving as cognitive “speed bumps” (47, 81).

The CHESS method was designed to mitigate bias in forensic psychiatric formulations. CHESS is the acronym of five sequential steps in the method: C, formulating **C**laim (preliminary opinion); H, establishing a **H**ierarchy of supporting evidence; E, examining the evidence for **E**xposure (in cross-examination); S, **S**tudying the evidence; and S, **S**ynthesizing a revised opinion. These steps can be repeated indefinitely until the psychiatrist is assured that his opinions are reasonable and logically sound, while still acknowledging possible weaknesses (48). The “SLOW” mnemonic is another cognitive forcing intervention that was made for general diagnostic reasoning, but still applicable in forensic psychiatric analysis. SLOW consists of S, “**S**ure about that? why?”; L, “**L**ook at the data? What is **L**acking? Does it **L**ink together?”; O, “What if the **O**pposite is true?”; and W, “**W**orst case scenario, what else could it be?” (85).

These interventions aimed at psychiatrists must be complemented by a conducive learning and/or working environment in order to provide favorable context for forensic psychiatric analysis (83). Senior and consultant psychiatrists should serve as good role models in clinical reasoning, especially by sharing their thought processes and their strategies to cope with uncertainty in forensic psychiatric analysis. The social and physical environment of the workplace should be designed to provide acceptable level of comfort to psychiatrist, prevent fatigue or burnout, and minimize interruptions or distractions. This would also include an effective management of forensic psychiatric practice to organize workload and reduce work-related stress.

## Conclusion

Clinical reasoning in the form of forensic psychiatric analysis is an essential process in a forensic psychiatric evaluation. It is needed in order to realize the aim of forensic psychiatry to provide a clear and objective explanation of an individual's mental state that is applicable to the legal question at hand. Forensic psychiatric analyses exhibit many parallel processes to clinical reasoning in general medicine. Consequently, the process can be elaborated through the lens of existing clinical reasoning theories such as the hypotheticodeductive model, illness script theory, and dual-process theory. These theories can also explain how a forensic psychiatrist's analysis can be influenced by case or contextual factors, leading to cognitive biases that shape their conclusions and recommendations.

A deeper understanding of analysis in forensic psychiatric assessments as a process of clinical reasoning brings practical benefit in forensic psychiatry and related fields. First, it may assist in analyzing the educational needs of psychiatrists and forensic psychiatrists. Drawing from extensive literature of clinical reasoning education, effective methods of teaching and learning forensic psychiatric analyses can be identified. Second, by realizing the potential pitfalls, training of debiasing strategies and other methods to minimize errors can be provided in residency and continuing professional development events.

## Author Contributions

NR and AR conducted literature searches and wrote the first draft of the manuscript. All authors contributed to manuscript revision, read, and approved the submitted version.

## Conflict of Interest

The authors declare that the research was conducted in the absence of any commercial or financial relationships that could be construed as a potential conflict of interest.

## Publisher's Note

All claims expressed in this article are solely those of the authors and do not necessarily represent those of their affiliated organizations, or those of the publisher, the editors and the reviewers. Any product that may be evaluated in this article, or claim that may be made by its manufacturer, is not guaranteed or endorsed by the publisher.
